# Pharmacological Therapy of Osteoporosis: A Systematic Current Review of Literature

**DOI:** 10.3389/fphar.2017.00803

**Published:** 2017-11-07

**Authors:** Vito Pavone, Gianluca Testa, Serena M. C. Giardina, Andrea Vescio, Domenico A. Restivo, Giuseppe Sessa

**Affiliations:** ^1^Dipartimento di Chirurgia Generale e Specialità Medico-Chirurgiche, Sezione di Ortopedia, A.O.U.P. Vittorio Emanuele, Università di Catania, Catania, Italy; ^2^Neurologic Unit, Department of Internal Medicine, Nuovo “Garibaldi” Hospital, Catania, Italy

**Keywords:** osteoroposis, pharmacological therapy, dual-energy x-ray absorptiometry, bisphosphonates, denosumab, estrogen, teriparatide

## Abstract

Osteoporosis is the most common bone disease affecting millions of people worldwide, particularly in elderly or in post-menopausal women. The pathogenesis is useful to understand the possible mechanism of action of anti-osteoporotic drugs. Early diagnosis, possible with several laboratory and instrumental tests, allows a major accuracy in the choice of anti-osteoporosis drugs. Treatment of osteoporosis is strictly related to severity of pathology and consists on prevention of fragility fractures with a correct lifestyle and adequate nutritional supplements, and use of pharmacological therapy, started in patients with osteopenia and history of fragility fracture of the hip or spine. The purpose of this review is to focus on main current pharmacological products to treat osteoporotic patients.

## Introduction

Osteoporosis, from the Greek term “porous bone,” is the most common bone disease, affecting millions of people worldwide. According to the World Health Organization, it is defined as a reduction in bone mineral density (BMD) of 2.5 standard deviations or more below that of the mean peak BMD of young adults when measured by dual-energy x-ray absorptiometry (World Health Organization, 2007). It is also characterized by microarchitectural deterioration of bone tissue, with a consequent increase in bone fragility and susceptibility to fracture, which results in high medical expenditures and substantial morbidity with a decrease in quality of life (Ray et al., 1997). Although osteoporosis risk factors differ by sex and age, osteoporotic fractures may result in substantial morbidity and mortality in both men and women (Halling et al., 2005). Approximately 34% of women, most of whom are post-menopausal, versus 17% of men are affected by this disease worldwide (Johnell and Kanis, 2006). Fractures in individuals over the age of 50 can be the first sign of weak bones from osteoporosis. In Europe, annually, 3.5 million new fragility fractures occur, the most common of which are in the spine (520,000 vertebral fractures), hip (610,000 hip fractures), and distal forearm (560,000 forearm fractures) (Hernlund et al., 2013). The economic burden of these injuries and prior injuries is estimated to be 37 billion. Incident fractures, long-term fracture care, and pharmacological prevention represent 66, 29, and 5% of this cost, respectively. Previous and incident fractures also account for 1,180,000 quality-adjusted life years lost during 2010. These costs are expected to increase by 25% in 2025 (Hernlund et al., 2013). Due to the rapid increase in disease burden and cost of osteoporosis worldwide, a reasonable goal of treatment is to focus on reducing fractures.

Although non-pharmacological treatments, such as weight bearing activity at least 30 min daily, smoking secession, and avoidance of heavy alcohol consumption, have important roles in maintaining bone health, pharmacological products play a key role in the treatment of osteoporosis and fracture prevention. The purpose of this review is to summarize the current primary pharmacological products used to treat osteoporosis.

## Pathogenesis

The etiopathogenesis of osteoporosis in post-menopausal women is primarily estrogen deficiency, which causes accelerated bone turnover, whereas in men and premenopausal women, vitamin D insufficiency and hyperparathyroidism are the primary causes. A combination of genetic, endocrine, and nutritional factors can alter the balance between bone resorption and deposition through the stimulation of osteoclast (bone-resorbing cells) activity and the inhibition of osteoblast and osteocyte (bone-forming cells) activity (Seeman and Delmas, 2006).

The primary endocrine factors involved in the development of osteoporosis are parathyroid hormone (PTH), vitamin D, calcitonin, and estrogen. PTH and vitamin D are directly connected: PTH can increase calcium absorption through kidneys, bone, and intestine; promote osteoclast activity; and activate vitamin D to form calcitriol, promoting calcium intestinal absorption. The roles of PTH and vitamin D are opposite to that of calcitonin, which binds to its receptor to reversibly block osteoclast function, thus blocking bone resorption. Estrogen can also block bone resorption by interacting with tissue-specific receptors, estrogen receptor α (ERα) and estrogen receptor β (ERβ), to increase osteoclast apoptosis, A decrease in estrogen production in post-menopausal women is one reason that this population has a higher incidence of osteoporosis.

Other factors contributing to bone resorption include physical factors, such as repeated bone micro-damage over time, which determines binding of RANKL (receptor activator of nuclear factor kappa-B ligand) to its receptor (RANK), which is expressed on pre-osteoclasts. The binding of RANKL to RANK results in the activation of osteoclasts. Additionally, oxidative stress causes the release of cytokines and prostaglandins that can increase osteoclastogenesis through up-regulation of RANKL and down-regulation of osteoprotegerin, a protein that normally blocks the binding of RANKL to RANK (Tabatabaei-Malazy et al., 2017).

## Diagnosis

Diagnosis of osteoporosis requires several laboratory and instrumental tests (Schweser and Brett, 2017). Patients should be prescreened starting at 50 years of age to maximize the benefit of fracture prevention (Gillespie and Morin, 2017). Laboratory tests are used to exclude secondary causes of the disease, such as thyroid and parathyroid dysfunctions and hypomagnesemia (Zheng et al., 2014; Naylor et al., 2016).

Dual-energy x-ray absorptiometry is the gold-standard diagnostic technique, providing a measure of BMD, as x-ray absorption is directly related to tissue calcium content (Compston et al., 2017). Osteoporosis is considered in patients with a T-score of -2.5 or less (Nayak and Greenspan, 2016). Limitations of dual-energy x-ray absorptiometry were reported in patients with previous fracture, osteoarthritis, osteomalacia, and metal implants; another disadvantage to this method is the propensity for discrepancy in collection and interpretation of results (Garg and Kharb, 2013).

Quantitative computed tomography can overcome some of the limitations of dual-energy x-ray absorptiometry, allowing a true measurement of bone density to be obtained with a single diagnostic device. However, quantitative computed tomography requires a higher radiation dose, is more expensive, and has poor quality control because of the need for computed tomography scanners to be calibrated for each measurement (Pisani et al., 2013).

The combination of FRAX score (Kanis et al., 2014; Bansal et al., 2015) and ultrasonography could be adopted in the diagnosis of osteoporosis, because it requires no radiation exposure and is cost-effective (Hoiberg et al., 2016; Karjalainen et al., 2016).

## Treatment

Treatment of osteoporosis is strictly related to severity of pathology. Initially, it is important to prevent fragility fractures with an active lifestyle and adequate nutritional supplements, including daily calcium and vitamin D intake, performing weight bearing activities, avoiding or stopping smoking, and avoiding heavy alcohol consumption (Pavone et al., 2015; Testa et al., 2015).

Depending on bone density, several pharmacological treatments could be used with the aim of increasing bone mass and strength by inhibiting bone resorption or promoting bone formation (Fukumoto and Matsumoto, 2017). In addition, surgical treatments such as vertebroplasty and kyphoplasty have been used for pain relief, but their benefits are still unclear (Watts et al., 2010).

Pharmacologic therapy should be initiated in patients with osteopenia; a history of fragility fracture of the hip or spi Pharmacologic therapy should be initiated in patients with osteopenia; patients with a T-score of -2.5 or lower in the spine, femoral neck, total hip, or 33% radius; and patients with a T-score between -1.0 and -2.5 if the FRAX^®^ 10-year probability for major osteoporotic fracture is ≥20% (Watts et al., 2010).

## Calcium or Vitamin D

Supplementation with calcium and vitamin D has a significant role in osteoporosis management, but is not sufficient to reduce fracture risk. The recommendations for dietary vitamin D intake are based on the benefits of the combination of calcium and vitamin D to skeletal health; there is no evidence supporting a benefit of vitamin D supplementation alone, although data has demonstrated utility in the prevention and treatment of glucocorticoid-induced osteoporosis (Hiligsmann et al., 2017). Generally, the recommended daily intake of calcium and vitamin D in post-menopausal osteoporotic women is 1200 mg (total intake by diet and supplements) or 800 international units. These values should be fixed before starting any pharmacological treatment for osteoporosis.

## Bisphosphonates

Oral bisphosphonates are efficacious and affordable and long-term safety data are available for most compounds. For these reasons, in the absence of specific contraindications, oral bisphosphonates are considered first-line pharmacological therapy for most post-menopausal women at high risk for fracture (Pazianas and Abrahamsen, 2016). Bisphosphonates act by interfering with specific intracellular pathways in osteoclasts, resulting in cellular toxicity. Specifically, they bind to hydroxyapatite and are thus absorbed by bone, inhibiting osteoclastic bone resorption via several modalities: cytotoxic or metabolic injury of mature osteoclasts, inhibition of osteoclast attachment to bone, inhibition of osteoclast differentiation or recruitment, and interference with osteoclast structural features necessary for bone resorption (i.e., components of the cytoskeleton) (Nayak and Greenspan, 2016).

There are two subclasses of bisphosphonates: nitrogen-containing bisphosphonates (NBPs; e.g., alendronate, ibandronate, pamidronate, risedronate, and zoledronate), which are the most common, and non-nitrogen-containing bisphosphonates (NNBPs; e.g., etidronate). NBPs inhibit the mevalonate pathway, a fundamental metabolic pathway involved in osteoclast formation and function; NNBPs act through the formation of metabolites that form toxic ATP analogs that induce osteoclast apoptosis (Garg and Kharb, 2013). Before initiating therapy, it is important to treat any comorbid conditions, such as hypocalcemia, vitamin D deficiency, and renal impairment. These conditions can be identified by measuring serum calcium, 25-hydroxyvitamin D (25[OH]D), and creatinine, respectively.

The first choice of bisphosphonate therapy is usually an oral regimen of alendronate or risedronate taken once a week on an empty stomach first thing in the morning with at least 240 mL of water because they are poorly absorbed *per os*. After administration, the patient should stand upright for at least 30–60 min and refrain from consuming food, drink, medications, or supplements for at least 30 min. In this way, assimilation and potential gastrointestinal adverse events are minimized (Nayak and Greenspan, 2016). The typical dosage is 10 mg daily (or 70 mg once-weekly) and 5 mg daily (or 35 mg once-weekly) of alendronate in tablet form for the treatment and prevention of osteoporosis, respectively. Intravenous administration of zoledronic acid (infused at least for 15 min yearly) or ibandronate (every 3 months as a 15- to 30-s intravascular injection) is suggested for patients in whom bisphosphonate use is contraindicated, such as those with low tolerance, gastrointestinal disease, or assimilation problems. Bisphosphonates should be initiated 4–6 weeks after a fracture and should not be discontinued in patients with an osteopathic fragility fracture who have been receiving the drug for less than 5 years, due to the potential for delayed healing time.

The most common adverse events, particularly for oral bisphosphonates, are Barrett’s esophagus and gastrointestinal disturbances such as dyspepsia, esophagitis, and esophageal varices. Rarely, atrial fibrillation and renal failure may occur. Therefore, intravascular bisphosphonates should not be used in patients with chronic kidney disease and an estimated glomerular filtration rate < 30–35 mL/min. Moreover, atypical femur fractures, especially subtrochanteric and diaphyseal fractures, have been linked to bisphosphonate use, likely due to over-suppression of bone turnover (Pazianas and Abrahamsen, 2016). Lastly, NBPs, but not NNBPs, have been associated with bisphosphonate-related osteonecrosis of the jaw, an oral complication that could arise in patients, especially those with recent maxillo-facial or oral surgery (Garg and Kharb, 2013).

## Denosumab

Denosumab is the first fully human monoclonal antibody that binds specifically to human RANKL to inhibit osteoclast formation and activation, thus inhibiting bone resorption. Indeed, this inhibition can stop the progression of bone erosion and loss (Takeuchi et al., 2016). Denosumab was approved for the treatment of post-menopausal osteoporosis because of its strong efficacy in reducing spine and hip fractures. It can be administered once every 6 months and it suppresses bone resorption by 80–90% (Suzuki et al., 2017). Denosumab is not used as a first-line treatment for osteoporosis, but it can be used as a first-line pharmacological treatment in certain patients who are intolerant to oral bisphosphonates or who have renal failure, a serious contraindication for bisphosphonate therapy that can lead to toxicity due to lack of renal clearance of the drug. The beneficial effects of denosumab are not observed until 1 month after initiation of therapy and its anti-resorptive effects last only 4–6 months, thereby providing a margin of safety in terms of total suppression of remodeling. Similar to bisphosphonates, hypocalcemia and vitamin D deficiency should be managed before starting and during treatment with denosumab (Pazianas and Abrahamsen, 2016). In clinical trials, denosumab was well-tolerated and did not cause jaw osteonecrosis, arterial fibrillation, or symptomatic hypocalcemia.

Denosumab should be used only in select patients. It is not recommended for premenopausal women or children, or as preventive therapy for osteoporosis; it should not be used in combination with other pharmacological agents for osteoporosis. Because denosumab inhibits the binding of RANKL to RANK, which is expressed on T-lymphocytes, B-lymphocytes, and dendritic cells in addition to pre-osteoclasts, an increased risk for infection has been reported as an adverse event of denosumab in several studies. Specifically, more frequent episodes of urinary tract infections in first-year kidney transplant recipients have been reported (Bonani et al., 2016). Therefore, antibiotic prophylaxis may be considered in patients with past recurrent infections, and patients should be instructed to report any signs of infection for appropriate treatment.

## Estrogen Replacement and Selective Estrogen Receptor Modulators

Because of the roles estrogen receptor α and estrogen receptor β play in osteoclast apoptosis, the use of estrogen replacement therapy or estrogenprogestin (hormone) replacement therapy with tibolone is effective for prevention of osteoporosis in post-menopausal women. Many studies show changes in lumbar spine, total hip, and femoral neck BMD; specifically, treatment with hormone replacement therapy increases bone density at the lumbar spine and reduced bone turnover markers at 2 years treatment (Cartwright et al., 2016). Because of a potential increased risk for venous thromboembolic disorders, breast cancer, cardiac events, stroke, and endometrial cancer, estrogen replacement is not recommended as first-line preventive treatment for osteoporosis and when it is initiated, it should be administrated at the lowest effective dose for a short period of time (Tabatabaei-Malazy et al., 2017). In fact, it has been reported that many women who abruptly stopped hormone replacement therapy were at a greater risk for incurring osteoporotic fractures (Lobo et al., 2016).

Selective estrogen receptor modulators are non-steroidal synthetic drugs with similar effects on bone and the cardiovascular system as estrogen, but without any of the adverse events on breast and endometrium. The most frequently used selective estrogen receptor modulators for the prevention of osteoporosis in post-menopausal women are raloxifene, lasofoxifene, and bazedoxifene, a recently FDA-approved drug. These drugs are typically used in combination with conjugated estrogens (Qaseem et al., 2017). Selective estrogen receptor modulators reduce vertebral fractures in osteoporotic women by increasing trabecular bone mass in the axial skeleton, but there is no statistically significant data demonstrating that they decrease the risk for non-vertebral or hip fractures compared to placebo. Furthermore, raloxifene was shown to increase cortical porosity (Börjesson et al., 2016). Selective estrogen receptor modulators are effective in the prevention and treatment of breast cancer in premenopausal women, but increase the rates of stroke, thromboembolism, leg cramps, and vasomotor symptoms in post-menopausal women (Tabatabaei-Malazy et al., 2017). For this reason, they are contraindicated for prevention or treatment of osteoporosis in premenopausal women, but they are suggested as first-line therapy for the prevention of osteoporosis in post-menopausal women.

## Calcitonin

Calcitonin inhibits bone resorption by increasing osteoblast activity. Until recently, calcitonin was considered a second line therapy for osteoporosis in settings where first-line drugs were intolerable or did not elicit a therapeutic response. To date, data on the effect of calcitonin on BMD of other skeletal sites are conflicting, as shown in recent studies. Calcitonin is available in injectable and intranasal; oral formulations, which are more convenient than other administration modalities, are in development (Bandeira et al., 2016). Women treated with calcitonin experience an increase in lumbar spine BMD and a decrease in biomarkers of bone turnover, especially women taking the oral formulation; however, calcitonin does not prevent new vertebral, non-vertebral, or hip fractures. Similarly, a recent major clinical trial failed to show that calcitonin is efficacious in preventing fractures (Henriksen et al., 2016).

## Teriparatide

Teriparatide it is a recombinant human parathyroid hormone, namely a peptide of PTH. It is the first, and currently the only, approved anabolic agent for the treatment of osteoporosis that stimulates osteoblastic bone formation to improve bone quality and bone mass (Lindsay et al., 2016). It activates osteoblasts by binding to PTH/PTHrP type 1 receptor, directly stimulating bone formation on active remodeling sites and on previously inactive bone surfaces, and initiating new remodeling sites. Several studies have shown a rapid rise in biochemical markers of bone formation during the first months of teriparatide treatment without an accompanying increase in bone resorption. Therefore, it stands to reason that in the early stages of treatment, bone formation exceeds bone resorption. Teriparatide causes an increase in bone density that is clearly observed on dual-energy x-ray absorptiometry, especially in the lumbar spine and femoral neck, where BMD values increase significantly, reducing fracture risk, as shown after 24 months of treatment, which is the total treatment duration approved for patients with osteoporosis at high risk for fracture.

### Strontium Ranelate

Strontium ranelate is an anti-resorptive agent approved in Europe for the treatment of men and post-menopausal women with severe osteoporosis who cannot tolerate other pharmacological agents. The mechanism of action is not entirely clear, but a modest antiresorptive effect has been noted, resulting from inhibition of osteoclast function and promotion of osteoblast differentiation and proliferation through the calcium sensing receptor (CaSR). This results in increased BMD, although this is not strictly related to a large reduction in fracture risk (Italian Society of Osteoporosis, Mineral Metabolism and Skeletal Diseases (SIOMMMS) et al., 2013). Common adverse events are cardiovascular events, venous thromboembolism, myocardial infarction, gastrointestinal discomfort, and signs and symptoms of nervous system disorders, such as headache, seizure, and memory loss. A rarely reported adverse event is allergic reactions, such as drug rash with eosinophilia and systemic symptoms (DRESS syndrome) (Kanis et al., 2011; Das and Crockett, 2013 ; Komm et al., 2015). Because of the high risk for heart injuries, strontium ranelate is now considered a second line treatment for osteoporosis, only used when other medications for osteoporosis are unsuitable, in the absence of contraindications. Additional measures, including restrictions in patients with heart or circulatory problems, are also recommended to minimize the cardiovascular risks (O’Donnell et al., 2006). The use of strontium ranelate alone or in combination for the treatment of osteoporosis has several limitations because of its potential adverse events when used long-term, but represents a valid available option for treating osteoporosis in selected patients.

### Guidelines and Doses

For patients at high risk for fracture, initial therapy should include alendronate, risedronate, zoledronic acid, and denosumab, which are approved agents with efficacy in reducing hip, non-vertebral, and spine fractures. Intravenous administration of teriparatide, denosumab, or zoledronic acid may be an appropriate initial therapy for patients unable to use oral therapy. Raloxifene or ibandronate should be considered in special cases where patients require drugs with spine-specific efficacy. Combination therapy is not generally recommended, but may be considered if a patient with high risk for fractures is already under treatment with estrogen for menopausal symptoms or raloxifene to reduce the risk of breast cancer; in these cases, an additional agent such as a bisphosphonate, denosumab, or teriparatide may be appropriate (**Table 1**) (Camacho et al., 2016).

**Table 1:** Brief classification of osteoporosis drugs, action mechanism and dosages (Camacho et al., 2016).

Drug class	Action mechanism	Drug	Dose
**Nutritional supplements**	Prevention of bone loss maintaining the normal level of calcium	Calcium and vitamin D	1200 mg daily intake
**Antiresorptive**			
Bisphosphonates	Cytotoxic or metabolic injury of mature osteoclasts; inhibition of osteoclast attachment to bone; inhibition of osteoclast differentiation; interference with osteoclast structural features necessary for bone resorption	Alendronate	10 mg per os daily 70 mg per os weekly
		Ibandronate	2.5 mg per os daily 150 mg per os monthly 3 mg intravenous every 3 months
		Risedronate	5 mg per os daily 35 mg per os weekly 150 mg per os monthly
		Zoledronic acid	5 mg intravenous once yearly
RANKL antibodies	Block of the binding of RANKL to RANK; osteoclast inactivation, apoptosis, and reduction in osteoclasts’ differentiation	Denosumab	60 mg subcutaneous every 6 months
SERMs	Interaction with bone’s estrogen receptors, increasing trabecular bone mass	Raloxifene	60 mg per os daily
Calcitonin	Increasing osteoblast activity	Calcitonin	200 IU intranasal once daily 100 IU subcutaneous qod
**Anabolic**			
PTH peptides	Activation of osteoblasts’ function by binding to PTH/PTHrP type 1 receptor	Teriparatide	20 μg subcutaneous daily


A recent study of Hambli et al. (2016) showed the ability of a computer model in performing a simulation on the effects of drug treatments and doses on bone volume. This represents a new frontier of prediction of the optimal treatment strategy, allowing a personalization of drug dosing and duration of treatment for a specific patient.

## Conclusion

Pharmacological treatment of osteoporosis is necessary to reduce the risk of fractures in older patients. An early diagnosis and characterization of the pathology aids in the choice of safe and effective anti-osteoporosis agents of those that are currently available. Oral bisphosphonates, with adequate supplementation of calcium and vitamin D, are considered the first choice for pharmacological therapy because of their efficacy and low costs. Newer pharmacological agents, such as teriparatide, denosumab and raloxifene, are likely to be used as second- or third-line treatments after an initial period of bisphosphonates, to increase BMD, suppress bone remodeling, and prevent possible atypical fractures. These agents have been shown to have beneficial effects on bone health, the fastest action times, and optimal beneficial durations of treatment and dosing requirements; however, due to high cost, their usage has been reserved for patients who are at high risk for fracture or who fail to respond to first-line treatment options. With further study and the identification of the primary genes and signaling pathways responsible for bone loss in individual patients, new treatment options will become available, allowing for the use of personalized therapy based on genetic risk and environmental factors.

## Author Contributions

All authors listed have made substantial, direct and intellectual contribution to the work, and approved it for publication. In particular, VP, GT and AV treated the orthopedic aspect, SG and DR treated pharmacological topic, GS revised the manuscript.

## Conflict of Interest Statement

The authors declare that the research was conducted in the absence of any commercial or financial relationships that could be construed as a potential conflict of interest.

## References

[B1] Bandeira L. Lewiecki E. M. Bilezikian J. P. (2016). Pharmacodynamics and pharmacokinetics of oral salmon calcitonin in the treatment of osteoporosis. *Exp. Opin. Drug Metab. Toxicol.* 12 681–689. 10.1080/17425255.2016.1175436 27070719

[B2] Bansal S. Pecina J. L. Merry S. P. Kennel K. A. Maxson J. Quigg S. (2015). US preventative services task force FRAX threshold has a low sensitivity to detect osteoporosis in women ages 50-64 years. *Osteoporos. Int.* 26 1429–1433. 10.1007/s00198-015-3026-0 25614141

[B3] Bonani M. Frey D. de Rougemont O. Mueller N. J. Mueller T. F. Graf N. (2016). Infections in de novo kidney transplant recipients treated with the RANKL inhibitor denosumab. *Transplantation* 10.1097/TP.0000000000001547 [Epub ahead of print]. 27798510

[B4] Börjesson A. E. Farman H. H. Movérare-Skrtic S. Engdahl C. Antal M. C. Koskela A. (2016). SERMs have substance-specific effects on bone, and these effects are mediated via ERαAF-1 in female mice. *Am. J. Physiol. Endocrinol. Metab.* 310 E912–E918. 10.1152/ajpendo.00488.2015 27048997 PMC4935145

[B5] Camacho P. M. Petak S. M. Binkley N. Clarke B. L. Harris S. T. Hurley D. L. (2016). American association of clinical endocrinologists and American college of endocrinology Clinical practice guidelines for the diagnosis and treatment of postmenopausal osteoporosis — 2016. *Endocr. Pract.* 22(Suppl. 4), 1–42. 10.4158/EP161435.GL 27662240

[B6] Cartwright B. Robinson J. Seed P. T. Fogelman I. Rymer J. (2016). Hormone replacement therapy versus the combined oral contraceptive pill in premature ovarian failure: a randomized controlled trial of the effects on bone mineral density. *J. Clin. Endocrinol. Metab.* 101 3497–3505. 10.1210/jc.2015-4063 27340881

[B7] Compston J. Cooper A. Cooper C. Gittoes N. Gregson C. Harvey N. (2017). UK clinical guideline for the prevention and treatment of osteoporosis. *Arch. Osteoporos.* 12:43. 10.1007/s11657-017-0324-5 28425085 PMC5397452

[B8] Das S. Crockett J. C. (2013). Osteoporosis- a current view of pharmacological prevention and treatment. *Drugs Des. Devel. Ther.* 7 435–448. 10.2147/DDDT.S31504 23807838 PMC3686324

[B9] Fukumoto S. Matsumoto T. (2017). Recent advances in the management of osteoporosis. *F1000Res.* 6:625. 10.12688/f1000research.10682.1 28529724 PMC5428497

[B10] Garg M. K. Kharb S. (2013). Dual energy X-ray absorptiometry: pitfalls in measurement and interpretation of bone mineral density. *Indian J. Endocrinol. Metab.* 17 203–210. 10.4103/2230-8210.109659 23776890 PMC3683192

[B11] Gillespie C. W. Morin P. E. (2017). Trends and disparities in osteoporosis screening among women in the United States, 2008-2014. *Am. J. Med.* 130 306–316. 10.1016/j.amjmed.2016.10.018 27884649

[B12] Halling A. Persson G. R. Berglund J. Johansson O. Renvert S. (2005). Comparison between the Klemetti index and heel DXA BMD measurements in the diagnosis of reduced skeletal bone mineral density in the elderly. *Osteoporos. Int.* 16 999–1003. 10.1007/s00198-004-1796-x 15605191

[B13] Hambli R. Boughattas M. H. Daniel J. L. Kourta A. (2016). Prediction of denosumab effects on bone remodeling: a combined pharmacokinetics and finite element modeling. *J. Mech. Behav. Biomed. Mater.* 60 492–504. 10.1016/j.jmbbm.2016.03.010 27026666

[B14] Henriksen K. Byrjalsen I. Andersen J. R. Bihlet A. R. Russo L. A. Alexandersen P. (2016). A randomized, double-blind, multicenter, placebo-controlled study to evaluate the efficacy and safety of oral salmon calcitonin in the treatment of osteoporosis in postmenopausal women taking calcium and vitamin D. *Bone* 91 122–129. 10.1016/j.bone.2016.07.019 27462009

[B15] Hernlund E. Svedbom A. Ivergård M. Compston J. Cooper C. Stenmark J. (2013). Osteoporosis in the European Union: medical management, epidemiology and economic burden. A report prepared in collaboration with the International Osteoporosis Foundation (IOF) and the European Federation of Pharmaceutical Industry Associations (EFPIA). *Arch. Osteoporos.* 8:136. 10.1007/s11657-013-0136-1 24113837 PMC3880487

[B16] Hiligsmann M. Neuprez A. Buckinx F. Locquet M. Reginster J. Y. (2017). A scoping review of the public health impact of vitamin D-fortified dairy products for fracture prevention. *Arch. Osteoporos.* 12:57. 10.1007/s11657-017-0352-1 28634891 PMC5486688

[B17] Hoiberg M. P. Rubin K. H. Hermann A. P. Brixen K. Abrahamsen B. (2016). Diagnostic devices for osteoporosis in the general population: a systematic review. *Bone* 92 58–69. 10.1016/j.bone.2016.08.011 27542659

[B18] Italian Society of Osteoporosis Mineral Metabolism and Skeletal Diseases (SIOMMMS) Italian Society of Rheumatology (SIR) Varenna M. Bertoldo F. Di Monaco M. Giusti A. (2013). Safety profile of drugs used in the treatment of osteoporosis: a systematical review of the literature. *Reumatismo* 65 143–166. 10.4081/reumatismo.2013.143 24192561

[B19] Johnell O. Kanis J. A. (2006). An estimate of the worldwide prevalence and disability associated with osteoporotic fractures. *Osteoporos. Int.* 17 1726. 10.1007/s00198-006-0172-4 16983459

[B20] Kanis J. A. Johansson H. Oden A. Cooper C. McCloskey E. V. Epidemiology and Quality of Life Working Group of IOF (2014). Worldwide uptake of FRAX. *Arch. Osteoporos.* 9:166. 10.1007/s11657-013-0166-8 24420978

[B21] Kanis J. A. Johansson H. Oden A. McCloskey E. V. (2011). A meta-analysis of the effect of strontium ranelate on the risk of vertebral and non-vertebral fracture in postmenopausal osteoporosis and the interaction with FRAX^®^. *Osteoporos. Int.* 22 2347–2355. 10.1007/s00198-010-1474-0 21287148

[B22] Karjalainen J. P. Riekkinen O. Toyras J. Jurvelin J. S. Kroger H. (2016). New method for point-of-care osteoporosis screening and diagnostics. *Osteoporos. Int.* 27 971–977. 10.1007/s00198-015-3387-4 26556741

[B23] Komm B. S. Morgenstern D. Yamamoto L. A. Jenkins S. N. (2015). The safety and tolerability profile of therapies for the prevention and treatment of osteoporosis in postmenopausal women. *Exp. Rev. Clin. Pharmacol.* 8 769–784. 10.1586/17512433.2015.1099432 26482902

[B24] Lindsay R. Krege J. H. Marin F. Jin L. Stepan J. J. (2016). Teriparatide for osteoporosis: importance of the full course. *Osteoporos. Int.* 27 2395–2410. 10.1007/s00198-016-3534-6 26902094 PMC4947115

[B25] Lobo R. A. Pickar J. H. Stevenson J. C. Mack W. J. Hodis H. N. (2016). Back to the future: hormone replacement therapy as part of a prevention strategy for women at the onset of menopause. *Atherosclerosis* 254 282–290. 10.1016/j.atherosclerosis.2016.10.005 27745704

[B26] Nayak S. Greenspan S. L. (2016). Cost-effectiveness of osteoporosis screening strategies for men. *J. Bone. Miner. Res.* 31 1189–1199. 10.1002/jbmr.2784 26751984 PMC4891297

[B27] Naylor K. E. Jacques R. M. Paggiosi M. Gossiel F. Peel N. F. McCloskey E. V. (2016). Response of bone turnover markers to three oral bisphosphonate therapies in postmenopausal osteoporosis: the TRIO study. *Osteoporos. Int.* 27 21–31. 10.1007/s00198-015-3145-7 25990354

[B28] O’Donnell S. Cranney A. Wells G. A. Adachi J. D. Reginster J. Y. (2006). Strontium ranelate for preventing and treating postmenopausal osteoporosis. *Cochrane Database Syst. Rev.* 4:CD005326. 10.1002/14651858.CD005326.pub3 16856092

[B29] Pavone V. Testa G. Alberghina F. Lucenti L. Sessa G. (2015). The importance of a correct diet in preventing osteoporosis. *J. Osteopor. Phys. Act* 3:160 10.4172/2329-9509.1000160

[B30] Pazianas M. Abrahamsen B. (2016). Osteoporosis treatment: bisphosphonates reign to continue for a few more years, at least? *Ann. N. Y. Acad. Sci.* 1376 5–13. 10.1111/nyas.13166 27525578

[B31] Pisani P. Renna M. D. Conversano F. Casciaro E. Muratore M. Quarta E. (2013). Screening and early diagnosis of osteoporosis through X-ray and ultrasound based techniques. *World J. Radiol.* 5 398–410. 10.4329/wjr.v5.i11.398 24349644 PMC3856332

[B32] Qaseem A. Forciea M. A. McLean R. M. Denberg T. D. Clinical Guidelines Committee of the American College of Physicians (2017). Treatment of low bone density or osteoporosis to prevent fractures in men and women: a clinical practice guideline update from the american college of physicians. *Ann. Intern. Med.* 166 818–839. 10.7326/M15-1361 28492856

[B33] Ray N. Chan J. Thamer M. Melton L. J. III (1997). Medical expenditures for the treatment of osteoporotic fractures in the United States in 1995: report from the National Osteoporosis Foundation. *J. Bone Miner. Res.* 12 24–35. 10.1359/jbmr.1997.12.1.24 9240722

[B34] Schweser K. M. Brett D. C. (2017). Osteoporosis: a discussion on the past 5 years. *Curr. Rev. Musculoskelet Med.* 10 265–274. 10.1007/s12178-017-9410-y 28389970 PMC5435641

[B35] Seeman E. Delmas P. D. (2006). Bone quality–the material and structural basis of bone strength and fragility. *N. Engl. J. Med.* 354 2250–2261. 10.1056/NEJMra053077 16723616

[B36] Suzuki T. Nakamura Y. Kato H. (2017). Changes of bone-related minerals during denosumab administration in post-menopausal osteoporotic patients. *Nutrients* 9:E871. 10.3390/nu9080871 28805705 PMC5579664

[B37] Tabatabaei-Malazy O. Salari P. Khashayar P. Larijani B. (2017). New horizons in treatment of osteoporosis. *Daru* 25:2. 10.1186/s40199-017-0167-z 28173850 PMC5297185

[B38] Takeuchi T. Tanaka Y. Ishiguro N. Yamanaka H. Yoneda T. Ohira T. (2016). Effect of denosumab on Japanese patients with rheumatoid arthritis: a dose–response study of AMG 162 (Denosumab) in patients with RheumatoId arthritis on methotrexate to Validate inhibitory effect on bone Erosion (DRIVE)—a 12-month, multicentre, randomised, double-blind, placebo-controlled, phase II clinical trial. *Ann. Rheum. Dis.* 75 983–990. 10.1136/annrheumdis-2015-208052 26585988 PMC4893103

[B39] Testa G. Pavone V. Mangano S. Riccioli M. Arancio A. Evola F. R. (2015). Normal nutritional components and effects on bone metabolism in prevention of osteoporosis. *J. Biol. Regul. Homeost. Agents* 29 729–736. 26403414

[B40] Watts N. B. Bilezikian J. P. Camacho P. M. Greenspan S. L. Harris S. T. Hodgson S. F. (2010). American association of clinical endocrinologists medical guidelines for clinical practice for the diagnosis and treatment of postmenopausal osteoporosis. *Endocr. Pract.* 16(Suppl. 3), 1–37. 10.4158/EP.16.S3.1 PMC4876714 21224201

[B41] World Health Organization (2007). *WHO Scientific Group on the Assessment of Osteoporosis at Primary Health Care Level: Summary Meeting Report; May5–7, 2004 Brussels, Belgium*. Geneva: World Health Organization, 1–17.

[B42] Zheng J. Mao X. Ling J. He Q. Quan J. Jiang H. (2014). Association between serum level of magnesium and postmenopausal osteoporosis: a meta-analysis. *Biol. Trace Elem. Res.* 159 8–14. 10.1007/s12011-014-9961-3 24728877

